# Ethylenediamine Salt Enhances the Solubility and Dissolution of Flurbiprofen

**DOI:** 10.1002/open.202300262

**Published:** 2024-01-12

**Authors:** Lei Gao, Xiaojie Li, Xiaolin Yan, Xianrui Zhang

**Affiliations:** ^1^ College of Food and Pharmaceutical Engineering Wuzhou University 543000 Wuzhou P. R. China

**Keywords:** flurbiprofen, ethylenediamine, crystal structure, thermal analysis, solubility

## Abstract

Drugs that are poorly soluble in water are difficult to absorb orally, resulting in low bioavailability. Flurbiprofen (FLU) is an arylpropionic acid nonsteroidal anti‐inflammatory drug belonging to BCS class II, with low water solubility. In this study, a novel flurbiprofen‐ethylenediamine salt (FLU‐EDA) was successfully prepared via solvent crystallization. Its crystal structure was determined via single‐crystal X‐ray diffraction (SXRD). Further, the physicochemical properties of FLU‐EDA salt were characterized by powder X‐ray diffraction (PXRD), differential scanning calorimetry (DSC), and Fourier transform infrared spectroscopy (FT‐IR). The solubility and intrinsic dissolution rate (IDR) of FLU‐EDA salt in water were investigated. The results showed that compared with FLU, the solubility and IDR of FLU‐EDA salt increased by 57‐fold and 32‐fold, respectively. This indicates that FLU‐EDA salt can significantly enhance the solubility and dissolution rate of flurbiprofen in water. This study provides basic data and theory for the development of new formulations of flurbiprofen.

## Graphical abstract

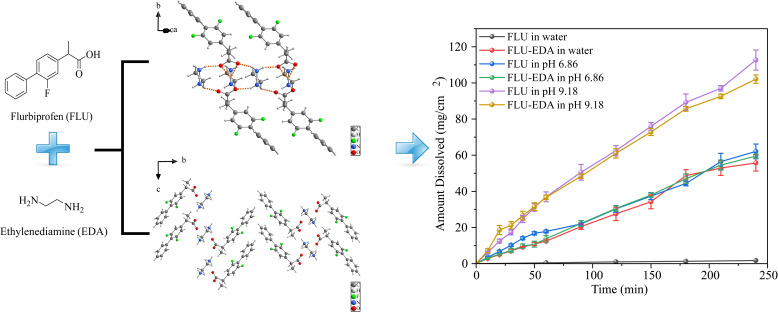

## Introduction

Drugs with poor solubility or a slow rate of dissolution are poorly absorbed in the gastrointestinal tract. Thus, insufficient drug concentrations fail to exhibit therapeutic effect or achieve the intended therapeutic goal.[^1^, ^2^, ^3^, ^4^, ^5^, ^6^, ^7^, ^8^, ^9^, ^10^] Therefore, improving the solubility and dissolution rates of drugs can enhance their absorption in the gastrointestinal tract, thereby enhancing the therapeutic effect. Salt formation is generally used to improve the pharmaceutical properties of drugs by enhancing their solubility and dissolution. By altering the crystal structure and charge state of drugs, their bioavailability and therapeutic effects can be improved.[^11^, ^12^, ^13^, ^14^, ^15^, ^16^, ^17^, ^18^, ^19^, ^20^]

Flurbiprofen, also known by its chemical name 2‐(4‐(2‐methylpropyl)phenyl) propanoic acid, is a nonsteroidal anti‐inflammatory drug (NSAID). It has limited solubility in water and is primarily used for its analgesic, antipyretic, and anti‐inflammatory properties. It is commonly used to alleviate symptoms of mild‐to‐moderate headaches, toothaches, arthritis, and muscle pain. However, its poor water solubility interferes with its absorption, resulting in poor bioavailability.[^21^, ^22^, ^23^, ^24^, ^25^] Diethylenediamine (EDA) is a frequently utilized pharmaceutical excipient and binder.[^26^, ^27^] It has an LD50 value of 637–1850 mg/kg when administered orally to rats.^[28]^ This compound is classified as having low toxicity and is readily soluble in water. EDA has been shown to enhance the solubility and dissolution rates of several drugs, including dabrafenib,^[29]^ theophylline^[30]^ and letermovir.^[31]^

**Figure 1:**
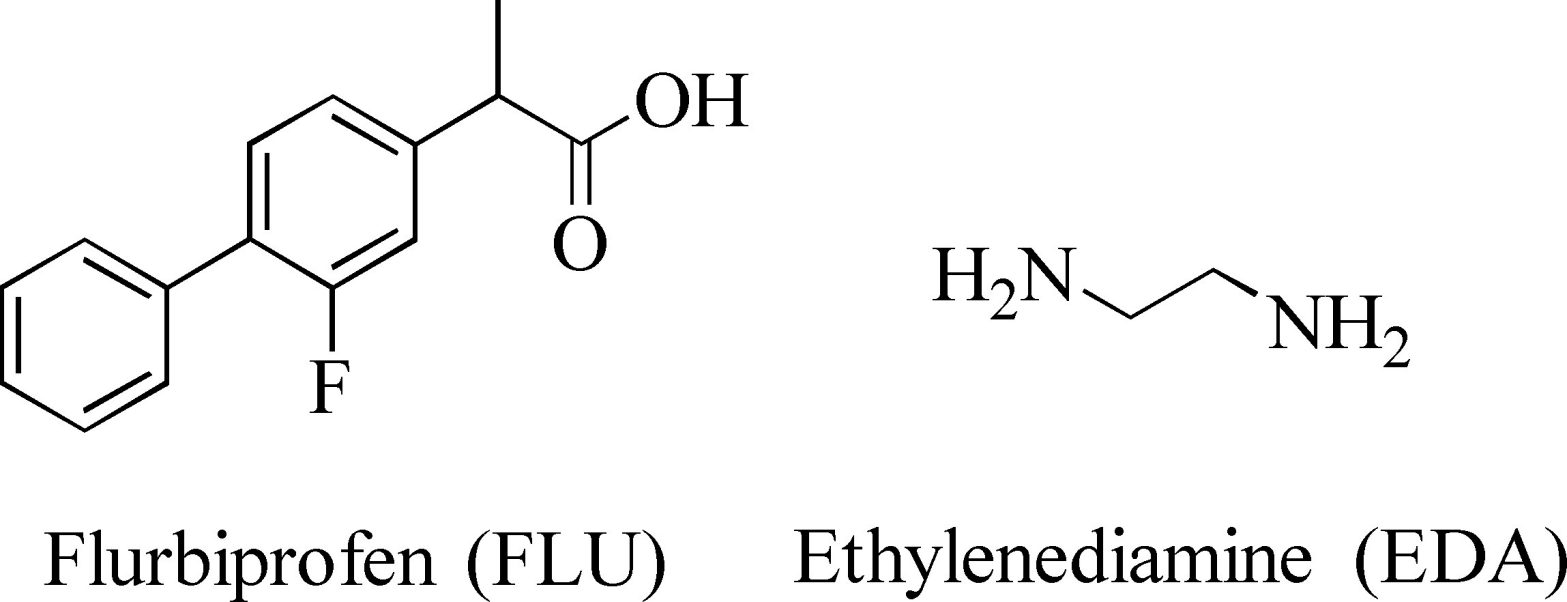
Molecular structures of FLU and EDA.

This study aimed to develop a novel flurbiprofen‐ethylenediamine salt with a 2 : 1 molar ratio. The crystal structure and physicochemical properties of this salt were determined. In addition, we investigated its solubility and intrinsic dissolution rate in water. The FLU‐EDA salt exhibited superior solubility and intrinsic dissolution rate compared with FLU. These findings provide valuable insights for further optimization of new formulations of flurbiprofen. The molecular structures of flurbiprofen and ethylenediamine are shown in Figure 1.

## Results and Discussion

### Crystal structure analyses

**Figure 2:**
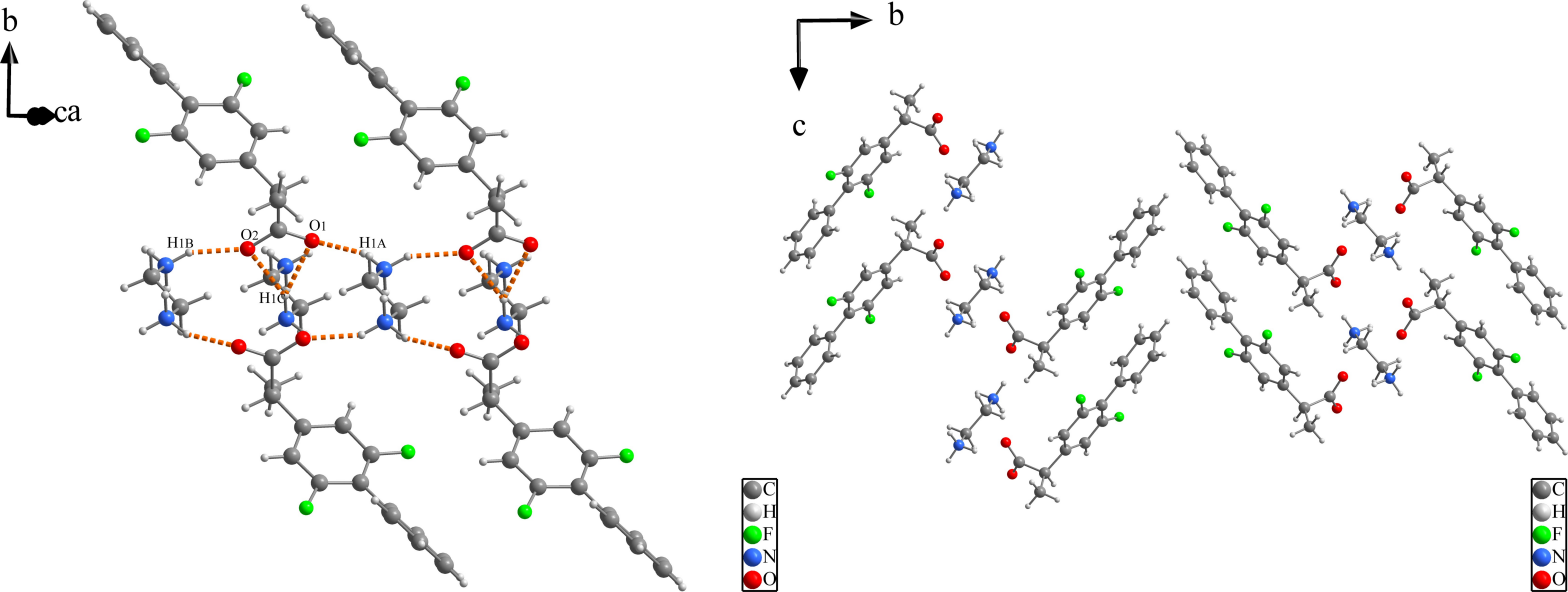
3D sandwich biscuit structures of FLU‐EDA salt through N^+^−H⋅⋅⋅O hydrogen bonds.

FLU‐EDA is a monoclinic crystal system with a P21/c space group. The FLU‐EDA consists of 1 FLU anion and 0.5 EDA divalent cation in the asymmetric unit. The FLU anion and EDA cations combine to form a three‐dimensional sandwich biscuit structure via N1^+^−H1A⋅⋅⋅O1, N1^+^−H1C⋅⋅⋅O1, N1^+^−H1B⋅⋅⋅O2, and N1^+^−H1C⋅⋅⋅O2 hydrogen bonds (Figure 2). The presence of two fluorine elements in the crystal is a result of disorder. The F1A has an occupancy rate of 0.4 and the F1B has an occupancy rate of 0.6. Tables 1 and 2 present the crystal structure parameters, hydrogen bond distances, and angles.

**Table 1:** Crystallographic parameters of FLU‐EDA salt.

	FLU‐EDA
chemical formula	C_15_H_12_FO_2_, 0.5 C_2_H_10_N_2_
formula sum	C_16_H_17_FNO_2_
formula weight	274.31
crystal system	monoclinic
space group	*P*21/*n*
*a* [Å]	6.0029(9)
*b* [Å]	38.509(4)
*c* [Å]	6.4454(7)
*α*[°]	90
*β*[°]	108.997(14)
*γ*[°]	90
Z	4
V [Å^3^]	1408.8(3)
D_calc_ [g cm^−3^]	1.293
M [mm^−1^]	0.094
R_1_ [I>2σ (I)]	0.1239
wR_2_ (all data, *F* ^2^)	0.2512
GOF	1.118
largest diff. peak and hole [e ⋅ Å^−3^]	0.551/−0.306
CCDC	2302144

**Table 2:** Hydrogen bond distances (Å) and angles (°) of FLU‐EDA salt.

H‐bond	d(D−H)	d(H⋅⋅⋅A)	d(D⋅⋅⋅A)	∠(DHA)
N1^+^−H1A⋅⋅⋅O1^[i]^	0.91	1.86	2.725(8)	157
N1^+^−H1C⋅⋅⋅O1^[ii]^	0.91	2.15	2.979(8)	151
N1^+^−H1B⋅⋅⋅O2^[iii]^	0.88	1.93	2.746(8)	155
N1^+^−H1C⋅⋅⋅O2^[ii]^	0.91	2.14	2.951(8)	147

Symmetry codes: [i] x−1, y, z−1; [ii] −x+2, −y, −z+2; [iii] x, y, z

Furthermore, despite our analysis of the FLU‐EDA crystal not yielding any type A or type B errors in the crystallography, it is worth noting that the R‐factor of the FLU‐EDA crystal is excessively high. Further in‐depth research reveals that the high R‐factor is caused by the disorderly vibration of the acetyl group in the FLU anion and the asymmetric orderliness of fluorine atoms. Additionally, the distance between the two C−O bonds in the carboxylic acid moiety of the FLU molecule ranges from 1.22 to 1.23 Å, further confirming the formation of a carboxylate.^[32]^ According to the ΔpKa rule, when the difference in pKa values between the main molecule and the ligand molecule is greater than 3, the substance formed is a salt; if the ΔpKa is less than 3, a cocrystal substance may be formed. The pKa value of FLU is 4.2,^[33]^ and the pKa value of EDA is 9.9.^[34]^ Considering that the ΔpKa value of FLU and EDA is greater than 3, it can be concluded that FLU‐EDA is indeed a salt.

### PXRD analyses

**Figure 3:**
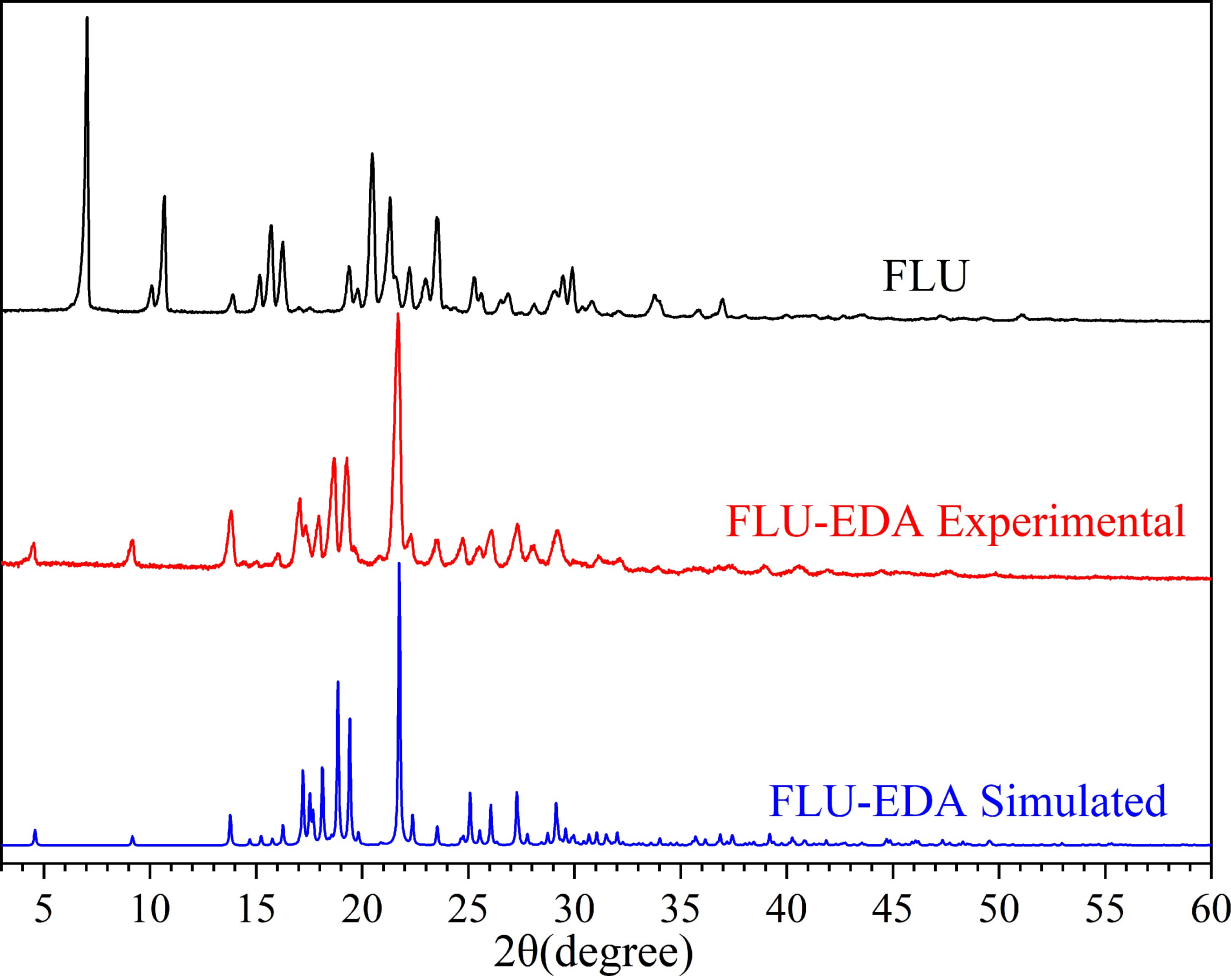
PXRD pattern of experimental FLU, experimental FLU‐EDA salt and simulated FLU‐EDA salt.

The FLU and FLU‐EDA salts were characterized using PXRD (Figure 3). The results showed that compared with FLU, the FLU‐EDA salt exhibited novel characteristic peaks at 4.52°, 9.16°, 13.84°, 17.08°, 17.96°, 18.70°, 19.28°, and 21.68°. The emergence of new peaks suggested the formation of a new solid‐state chemical form. Further, the experimental PXRD pattern of FLU‐EDA salt matched its simulated crystal structure pattern, indicating that high‐purity FLU‐EDA salt can be obtained via solution crystallization.

### Thermal analyses

**Figure 4:**
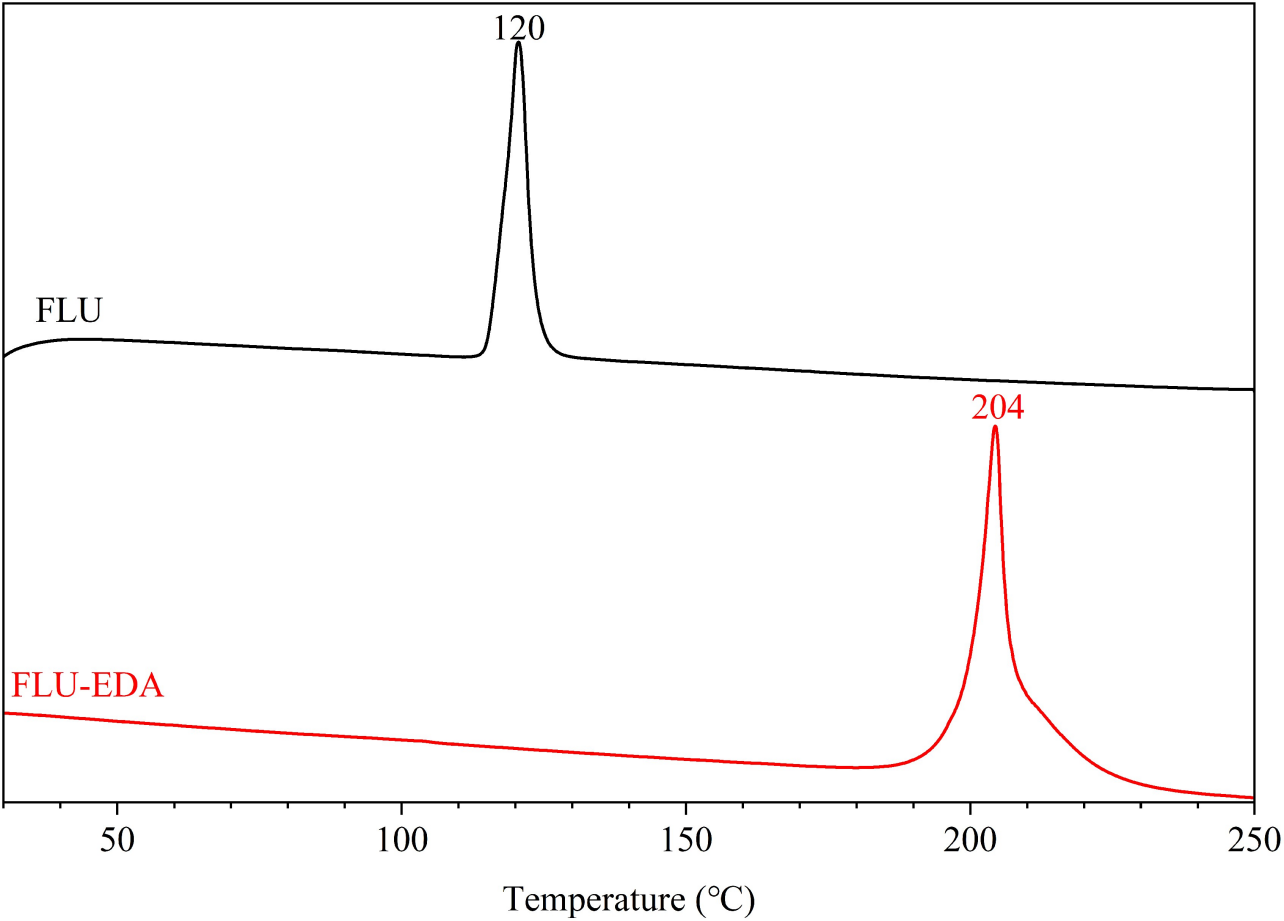
The DSC thermograms of FLU and FLU‐EDA salt.

The thermodynamic stability of FLU and FLU‐EDA salt was investigated via DSC (Figure 4). The FLU displayed an endothermal peak at 120 °C, whereas the FLU‐EDA salt exhibited an endothermal peak at 204 °C. Notably, the DSC endothermal peak of FLU‐EDA salt was higher than that of FLU, suggesting that FLU‐EDA salt exhibited enhanced thermodynamic stability.

### FT‐IR analyses

**Figure 5:**
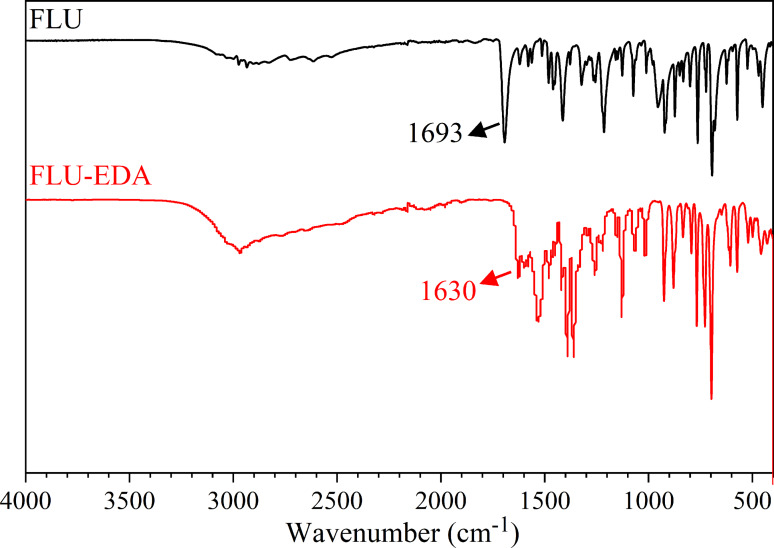
The Fourier‐transform infrared spectra of FLU and FLU‐EDA salt.

The infrared spectra of FLU and FLU‐EDA salt were compared, as shown in Figure 5. The carboxylic group in flurbiprofen serves as the hydrogen‐bonding site. The formation of the salt alters the stretching vibration peak of v(C=O). The stretching vibration peak of v(C=O) for flurbiprofen occurred at 1693 cm^−1^, whereas it occurred at 1630 cm^−1^ for FLU‐EDA salt. This discrepancy indicated the formation of a new substance. Notably, simple physical mixtures do not induce changes in the stretching vibration peak of v(C=O). The observed changes were primarily attributed to proton transfer and hydrogen bonds formation following salt formation.

### Solubility and Dissolution studies

**Figure 6:**
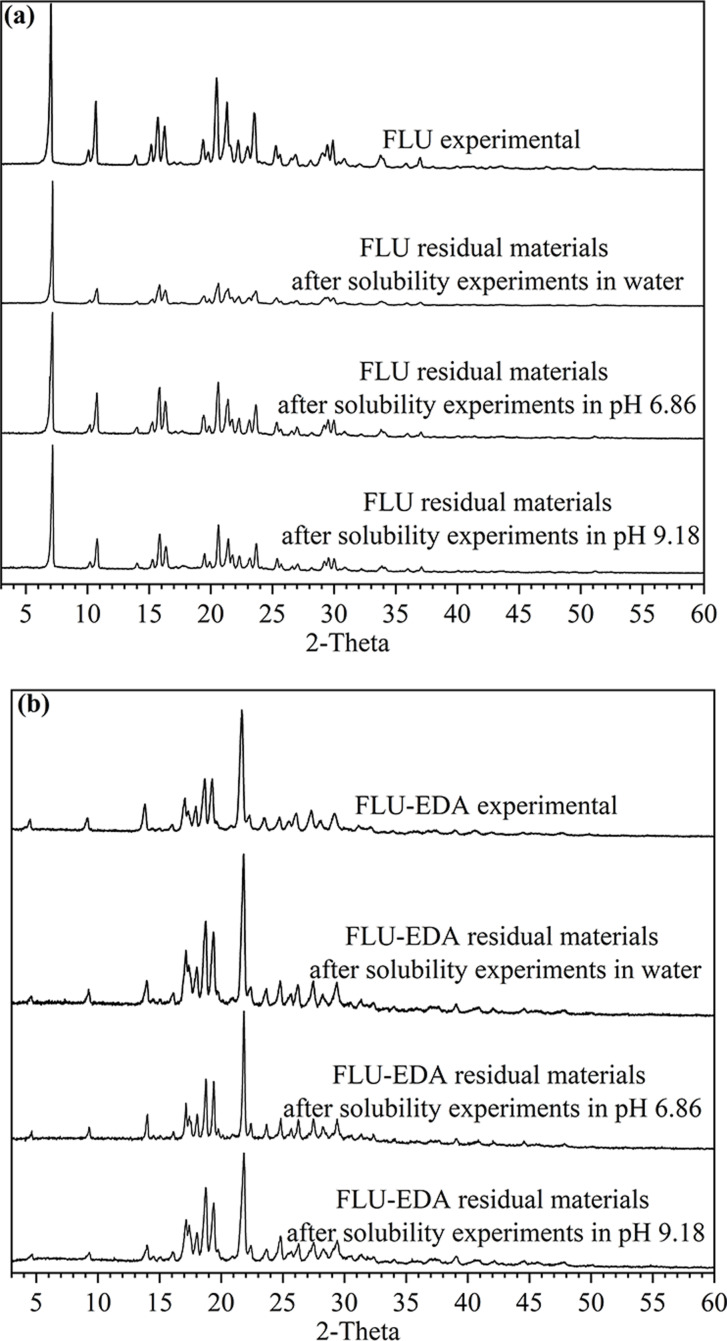
Comparison PXRD patterns of experimental and residual materials of FLU and FLU‐EDA salt in water, pH 6.86 and pH 9.18 buffer solutions.

The solubilities of FLU and FLU‐EDA salt in water, pH 6.86 buffer and sodium tetraborate buffer are shown in Table 3. The solubility of FLU‐EDA in water and pH 9.18 buffer solution higher than that of FLU, while in pH 6.86 buffer solution, the solubility of FLU was greater than that of FLU‐EDA. Compared with FLU, the solubility of the FLU‐EDA salt increased by 57‐fold and 1.17‐fold in water and pH 9.18 buffer solution, respectively. However, it decreased slightly in pH 6.86 phosphate buffer, which indicates that the FLU‐EDA salt has better solubility than FLU in water and alkaline environments. Further, the stability of residual samples were analyzed via PXRD. The results demonstrated sample stability under the three tested conditions (Figure 6).

**Table 3:** Measured solubility values of FLU and FLU‐EDA salt in water, pH 6.86 and pH 9.18 buffer solutions at 37 °C.

Medium	Compound	Concentration (mg/L)	IDR in water (mg/(cm^2^ ⋅ min))	Residue
water	FLU	26.04±1.33	0.0062	Stable
	FLU‐EDA	1489.73±104.11	0.2014	Stable
pH 6.86	FLU	3042.09±66.31	0.3366	Stable
	FLU‐EDA	2802.13±30.78	0.1955	Stable
pH 9.18	FLU	5549.66±101.27	0.6211	Stable
	FLU‐EDA	6473.14±99.37	0.5677	Stable

**Figure 7:**
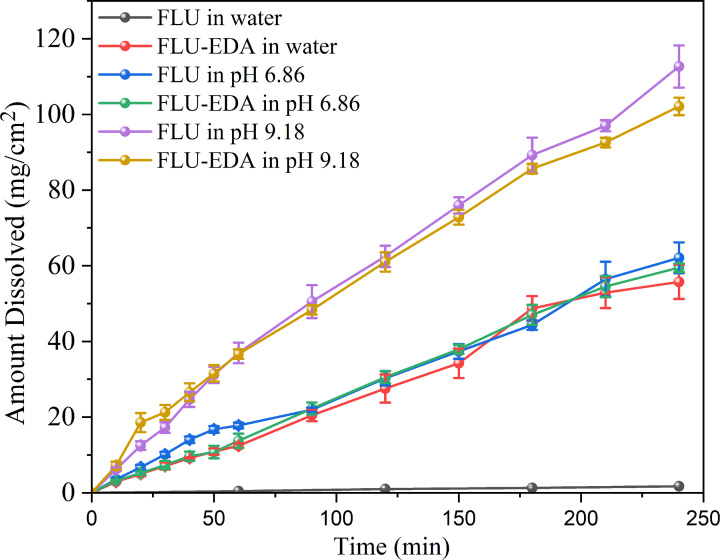
Dissolution profiles for FLU and FLU‐EDA salt in water, pH 6.86 and pH 9.18 buffer solutions.

We conducted a comprehensive study of the IDRs of FLU and FLU‐EDA salts under different conditions as mentioned above, and the results are shown in Figure 7 and Table 3. FLU‐EDA exhibited higher IDR values than FLU in water. However, the IDR of FLU‐EDA salt was slightly lower than that of FLU in the pH 6.86 and pH 9.18 buffer solution.

### Stability studies

**Figure 8:**
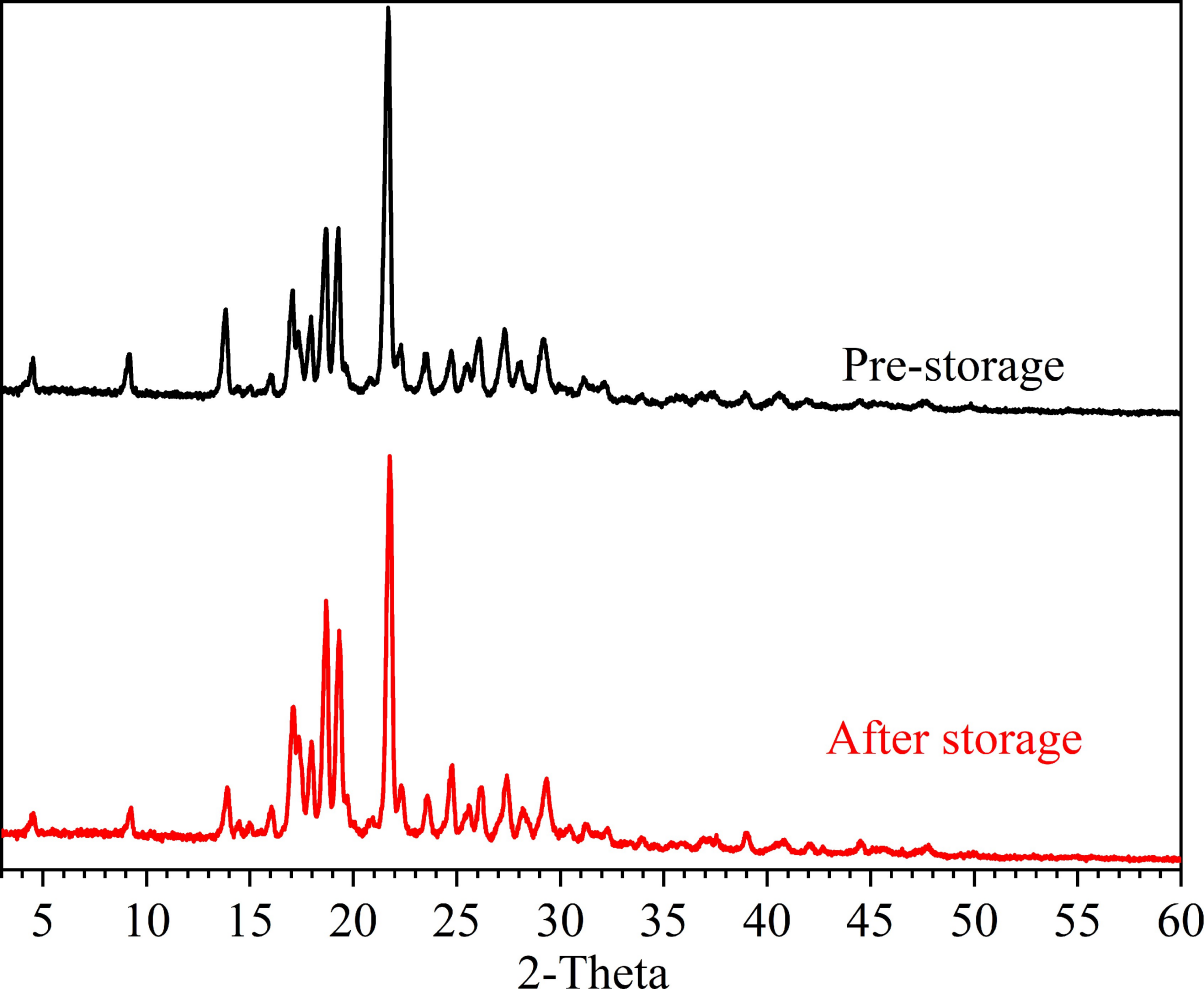
PXRD patterns of FLU‐EDA salt before and after storage under 75 % RH for 20 days.

The use of drug salts in solid dosage forms can lead to destabilization, particularly when the salt dissociates and negatively impacts the product performance. Therefore, it is essential to determine the instability of salts encountered in solid dosage forms in order to ensure the quality of the final product. In this study, the stability of FLU‐EDA salt was evaluated by storing the samples at 75 % relative humidity (RH) and 35 °C for 20 days. Subsequently, PXRD tests were conducted to assess the stability (Figure 8). The results revealed that FLU‐EDA salt remained stable in high humidity environments.

## Conclusions

Flurbiprofen, an NSAID belonging to arylpropionic acid class, is a BCS Class II drug due to its low water solubility. In this study, we enhanced the water solubility of flurbiprofen by introducing the pharmaceutical excipient ethylenediamine, resulting in the development of a new FLU‐EDA salt. The crystal structure of this salt was successfully solved via single‐crystal X‐ray diffraction (SXRD), suggesting that the asymmetric unit consisted of a single flurbiprofen anion and 0.5 ethylenediamine divalent cation. To further characterize the physicochemical properties of the FLU‐EDA salt, PXRD, DSC, and FT‐IR spectroscopy were employed. Additionally, we determined the solubility and IDR of the FLU‐EDA salt in water, pH 6.86 phosphate buffer, and pH 9.18 sodium tetraborate buffer solution. Notably, our results demonstrated an increase in the solubility and IDR of the FLU‐EDA salt in water. Overall, this study introduces a novel approach for flurbiprofen formulation by synthesizing the FLU‐EDA salt with enhanced water solubility. These findings have significant implications for the development of innovative flurbiprofen formulations.

## Experimental Section

### Instrumentations and materials

The study utilized the PNA X‐pert pro X‐ray powder diffractometer (PANalytical, Netherlands), Agilent Gemini E single crystal diffractometer (Agilent Technologies, USA), Thermo Nicolet iS50 Fourier Transform Infrared (FT‐IR) Spectrometer (Thermo Fisher Scientific, USA), NETZSCH DSC 200 scanning calorimeter (NETZSCH, Germany), PJ‐3 LAB Tablet Four‐Usage Tester (Tianjin, China), and UV‐2600 UV‐Vis spectrophotometer (Shimadzu, Japan). All chemical reagents and drugs utilized in the experiment were of analytical grade. The crystal structure of FLU‐EDA salt was determined using the SHELXS program and subsequently refined with the SHELXL program.[^35^, ^36^]

### Preparation of FLU‐EDA salt

Initially, 200 mg of FLU was completely dissolved in a 10 mL acetonitrile solvent. Next, 34 μL of ethylenediamine monohydrate, with a molar ratio of 2 : 1, was added dropwise to the solution. The mixture was stirred for 30 minutes, leading to the precipitation of a significant amount of white powder. Subsequently, the solution was filtered and left to dry for 24 hours. The product yield was 174 mg (75 %).

FLU‐EDA salt crystals were prepared by dissolving 20 mg of FLU‐EDA in a 6 mL mixture of ethanol and water (V_ethanol_ : V_water_=5 : 1). The mixture was stirred for 1 hour and then left to volatilize at room temperature. Massive colorless crystals wereobtained after approximately 15 to 20 days.

### Solubility and intrinsic dissolution rate studies

The solubility and intrinsic dissolution rates of FLU and FLU‐EDA salts were determined via UV spectrophotometry at a UV absorption wavelength of 247 nm.In a typical solubility experiment, an excess of sample was placed in a round‐bottomed flask containing 10 mL of medium. The flask was then stirred on a thermostatically heated magnetic stirrer at a temperature of 37±0.5 °C and 800 r/min speed for 24 hours. The supernatant was then collected and passed through a 0.22 μm filter membrane. The filtrate was used to determine the concentration using a UV spectrophotometer. Each experiment was performed in triplicate to obtain an average value.In a typical experiment to determine the intrinsic dissolution rate, a 100 mg sample was compressed under a pressure of 10 MPa to form a tablet with a surface area of 0.5 cm^2^. The periphery and bottom surface of the tablet were sealed with paraffin. The dissolution was performed at 50 rpm and a temperature of 37±0.5 °C, using 900 mL of dissolution medium. The concentration at different time points was measured using a UV spectrophotometer, with each experiment performed in triplicate to obtain the average value.

## Author Contributions

All authors conceptualized and designed the study. Material preparation and experimental study were performed by Xian‐Rui Zhang and Xiao‐Jie Li. The data analysis and software calculation were performed by Xiao‐Lin Yan and Lei Gao. The first draft of the manuscript was written by Lei Gao, and all authors comments on the previous version of the manuscript. All authors read and approved the final manuscript.

## Conflict of interests

The authors declare no conflict of interest.

1

## Data Availability

Research data are not shared.
